# Efficiency of mixed and rigid occlusal stabilization splints: Randomized clinical trial

**DOI:** 10.1590/1807-3107bor-2024.vol38.0017

**Published:** 2024-03-11

**Authors:** Layza Rossatto OPPITZ, Ana Carolina Mastriani ARANTES, Roberto Ramos GARANHANI, Carlos Alberto COSTA, Cristiano Miranda de ARAUJO, Orlando Motohiro TANAKA, Patricia Kern di Scala ANDREIS, Claudia SCHAPPO, Sérgio Aparecido IGNÁCIO, Aline Cristina Batista Rodrigues JOHANN, Rodrigo Nunes RACHED, Elisa Souza CAMARGO

**Affiliations:** (a) Pontifícia Universidade Católica do Paraná – PUCPR, School of Medicine and Life Sciences, Post-Graduate Program in Dentistry, Orthodontics, Curitiba, PR, Brazil.; (b) Zenith Educação Continuada, Department of Prosthetic Dentistry, Florianópolis, SC, Brazil.; (c) Universidade Tuiuti do Paraná, Postgraduate Program in Communication Disorders, Curitiba, PR, Brazil.; (d) Pontifícia Universidade Católica do Paraná – PUCPR, School of Medicine and Life Sciences, Undergraduate Program in Dentistry, Curitiba, PR, Brazil.

**Keywords:** Sleep Bruxism, Occlusal Splints

## Abstract

Occlusal stabilization splints are the most common treatment for controlling the deleterious effects of sleep bruxism. This study aimed to evaluate the effectiveness of a low-cost, mixed occlusal splint (MOS) compared to that of a rigid splint. A randomized clinical trial was performed on 43 adults of both sexes with possible sleep bruxism and satisfactory dental conditions. They were divided into rigid occlusal splint (ROS) (n = 23) and MOS (n = 20) groups. Masticatory muscle and temporomandibular joint (TMJ) pain intensity (visual analog scale), quality of life (WHOQOL-BREF), indentations in the oral mucosa, anxiety, and depression (HADS), number of days of splint use, and splint wear were evaluated. All variables were evaluated at baseline (T0), 6 months (T6), and 12 months (T12) after splint installation (T0), and splint wear was evaluated at T6 and T12. Student’s t-test, Mann–Whitney U test, non-parametric Friedman’s analysis of variance for paired samples and pairwise multiple comparisons, Pearson’s chi-square test, two-proportion z-test, non-parametric McNemar’s and Cochran’s Q, and Wilcoxon tests were used (p < 0.05). In both groups, there was a decrease in TMJ pain and pain intensity over time and improvements in the quality of life scores. At T6, there was a higher rate of splint wear in the MOS group than in the ROS group (p = 0.023). The MOS showed a higher rate of wear than the rigid splint but had similar results for the other variables. Therefore, the use of a mixed splint appears to be effective in controlling the signs and symptoms of sleep bruxism.

## Introduction

Sleep bruxism (SB) is a masticatory muscle activity^1^ affecting 8–31% of the adult population, regardless of sex. Disorders of the central nervous system may be involved in the etiology of SB,^1^ which may also be associated with genetic factors,^2^ the consumption of psychoactive drugs, pathological processes, medication use, stress, depression,^3^ and anxiety.^4^

SB can be characterized by severe signs, such as ground teeth, occlusal trauma, hypertrophy of masticatory muscles,^3^ and indentations of the buccal mucosa and tongue.^5^ Damage on periodontal tissue, dental hard tissues, dental restorations and implants, and musculoskeletal tissues can also be observed.^6^

There is no effective treatment to eliminate SB; therefore, the therapeutic approach is focused on preventing damage and treating its pathological effects on the masticatory system.^7^ Occlusal splints are the most popular method to prevent the consequences of SB.^8^ These allow the TMJ to adopt a more stable orthopedic joint position,^9^ which can reduce temporomandibular disorder (TMD) symptoms, allow for balanced occlusion, change afferent impulses for the central nervous system, improve the vertical dimension, correct the condylar position, and aid in cognitive awareness.^10^

Different models of occlusal stabilization splints have been used clinically.^9^Rigid splints are considered effective and are most commonly used.^12-13^ However, the cost is higher as they require the work of a dental technician if made according to the traditional method; for the manufacture of printed splints, software, and three-dimensional printers are needed.^14^ Soft splints are also popular; however, there is no evidence of their efficacy and effectiveness,^15^ and they seem to stimulate muscle contractions,^12^which can aggravate bruxism.^10,16^ The mixed splint,^10^ consisting of an acetate sheet and a self-curing acrylic cover, is built only by dental surgeons, which requires longer chair time. This can lead to a higher cost, considering the care in private practice. However, as far as public health services are concerned, clinical attendance time is not a limiting factor, given the benefits of the use of occlusal splints for the low-income population. Moreover, mixed splints have the advantage of reducing equipment expenses and the possibility of being made immediately after oral rehabilitation, which minimizes the risk of damage to prostheses and restorations;^10^however, there is no evidence of their efficiency in the literature.

The need for low-cost occlusal splints is a worldwide concern.^17^ In Brazil, the public health service provides specialized care in the areas of endodontics, periodontics, dentistry, surgery, and prosthetics.^16^Occlusal splints for the control of SB, despite the great demand, are not included in public policies, possibly because of their high cost.^14^

Thus, the present study aimed to evaluate the efficacy of a low-cost, mixed occlusal splint (MOS) compared to that of a rigid splint in patients with SB, considering its durability and effect on masticatory muscle and TMJ pain, quality of life (QoL), indentations in the oral mucosa, anxiety, and depression.

## Methodology

A randomized clinical trial was performed from 2017 to 2019 according to the CONSORT guidelines (Register 10.17605/OSF.IO/2XE6K). The study was approved by the University’s Ethics Committee (2.309.631) and all procedures were performed in compliance with the 1964 Declaration of Helsinki and its later amendments.

Two operators participated in this study; both dentists were trained to perform all procedures (examination, treatment, and evaluation) in a standardized manner.

### Blinding

It was not possible to blind the operators as it was necessary for the devices to be visually analyzed during the preparation, adjustment, and evaluation of the two types of splints.

### Sample

The sample size was calculated using the sampling proportions method for an infinite population with a confidence level of 95%, considering p = q = 50% as the proportion of favorable cases in the sample. As the number of variables in the questionnaires was large, a sample size of n = 60 was chosen, with n = 30 for each group and a maximum sampling error of 13.9%, with a sample size of n = 38 at T2, with a maximum sampling error of 16%.

Participants were selected from the University’s dental clinic. The inclusion criteria were adults (20–59 years) of both sexes with possible SB and satisfactory dental condition (no active periodontal disease, extensive caries, or insufficient teeth for device retention). The exclusion criteria were obstructive sleep apnea and/or frequent use of alcohol and narcotics (assessed using yes or no questions) and mouth-opening limitations impeding the necessary clinical procedures. Individuals who answered positively to at least one of the following two questions based on their behavior in the last 30 days were considered to have possible SB:^2^

1. Are you aware that you grind your teeth during sleep?
2. Did someone tell you that you grind your teeth in your sleep?

Individuals included in the study signed informed consent forms and were randomly assigned to the following groups: Rigid Occlusal Splint (ROS), a splint made by a dental surgeon and technician; and Mixed Occlusal Splint (MOS), which was made by a dental surgeon. Randomization was performed electronically using a simple randomization method in Microsoft Excel. The participant allocation list was stored in an opaque envelope and sealed after randomization.

All procedures were performed in person, with clinical examinations and questionnaires completed by the participants at the beginning (T0), 6 m (T6), and 12 m (T12) after the installation of the occlusal splints.

### Primary outcomes

#### Orofacial pain

Each patient was asked if they experienced pain in any region of the face, and if they answered “yes,” they pointed to that region. Thereafter, the operator performed light palpation (1 kgF) on the masseter and temporalis muscles and 0.5 KgF on the TMJ region, to confirm the location of the pain, and its presence in each of the three regions was recorded, whether unilateral or bilateral.

In addition, a visual analog scale was used, with “no pain” being the leftmost number (value = 0) and “worst pain” being the rightmost number (value = 10).^18^ Pain was classified as absent when the intensity was 0, mild from 1 to 2, moderate from 3 to 7, and severe from 8 to 10.

#### Quality of life

Participants answered the World Health Organization Quality of Life questionnaire (WHOQOL-BREF^20^), consisting of 26 questions, of which 24 were divided into four domains: physical, psychological, social relations, and environmental. The remaining two questions assess self-perceived QoL and QoL satisfaction with health. The maximum possible score was five points, and the scale used for its interpretation was: 1–2.9, “needs improvement” (0); 3–3.9, “regular” (1); 4–4.9, “good” (2); and 5, “very good” (3).

## Secondary outcomes

### Indentations in the oral mucosa

The occurrence of indentation was evaluated during the clinical examination. Upon minimal observation, indentations were considered to be present, according to the following criteria:

- *Tongue indentation*: This exam was performed with the tongue pulled and was present when there was notable formation of tongue indentations.^19^
- *Buccal indentation*: This was considered present when there was an indentation on the right, left, or both sides of the buccal mucosa.
- *Labial indentation*: The indentations of the upper and lower lip were assessed and considered present when observed in one or both lips.

### Anxiety and depression

The Hospital Anxiety and Depression Scale (HADS^22^) was used, which comprises 14 questions, seven related to anxiety and seven related to depression, distributed alternately.

Values from 0–7 indicated no anxiety/depression, 8–10 indicated mild anxiety/depression, 11–14 represented moderate anxiety/depression, and 15–21 indicated severe anxiety/depression.

## Occlusal splints

The splints were evaluated six and 12 months after installation and the following were recorded:

- The presence or absence of wear on the splint, assessed visually (presence of marks on the surface with loss of enamel)
- How many times a week the splint was used (time of use).
- When wear was observed on the splint, it was immediately repaired using a self-curing acrylic resin. Finishing and polishing were carried out in such a manner as to maintain the initial conformation.

## Construction of rigid occlusal splints

The participants had both dental arches molded with alginate (Jeltrate® Dustless, New York, USA), and a bite registration was taken in type 7 wax in maximum habitual intercuspation. The molds were cast with a type III stone plaster (Asfer, São Caetano do Sul, Brazil), and the models were sent to a dental technician along with the wax bite registration, who performed the assembly on a semi-adjustable articulator using the Camper Plan (Bio-Art, São Carlos, Brazil), defined the thickness of the splint on the incisal pin (approximately 2 mm in the region of the first posterior contact), and made rigid devices with thermo-polymerizable acrylic resin (Vipicril Plus, Pirassununga, Brazil).

## Construction of mixed occlusal splints

Molding of the maxillary arch was performed using alginate, and a stone plaster model was obtained. In the following consultation, splints were created based on the description proposed by Okeson,^10^ with the following changes, as suggested by the authors of the study:

- Using the Maxicut Drill (Series XXBL105, Tri Hawk, Morrisburg, Canada), all cusp tips were worn until the acetate splint was perforated in such a way that the acrylic resin to be placed came in direct contact with the teeth;
- A single rod was manufactured with self-curing acrylic resin (Jet, Classico, São Paulo, Brazil), which was placed on roughened surfaces previously moistened with a monomer. In the plastic phase of the resin, the device was placed in the maxillary arch and the patient was asked to occlude until the lower anterior teeth touched the height plane.

Both types of splints had anterior and lateral disocclusions in the canine region and simultaneous bilateral contact. During installation, all occlusal contacts were checked with carbon paper and wear was performed when adjustments were required. All participants were instructed to wear the splints during sleep for seven days a week and return for adjustments after 10, 30, and 60 days. If adjustments were required at any time, researchers were available.

## Statistical analysis

For comparisons between time points within each group, the non-parametric Wilcoxon test for paired samples, Friedman analysis of variance (ANOVA) non-parametric test for paired samples, Friedman’s pairwise multiple comparisons non-parametric test, and non-parametric McNemar’s and Cochran’s Q-tests were used.

In the evaluation of ordinal variables between times in both groups united, the non-parametric Wilcoxon test for paired samples was used. To assess the frequency of dichotomous variables, a non-parametric McNemar’s test was conducted.

Pearson’s chi-square test was used to assess the dependence between dichotomous nominal variables, followed by a z-test for differences between two proportions. The same tests were applied for indentations and pain between the three time points (0, 6, and 12 months) and for device wear between six and 12 months.

The Shapiro–Wilk’s normality test was performed for continuous variables. To compare the mean values according to the group, in the variables that followed a normal distribution, the parametric Student’s t-test was performed for independent samples, and in those that did not follow a normal distribution, the non-parametric Mann–Whitney U test for independent samples was used. The Mann–Whitney U test for independent samples was used to compare ordinal variables according to the group.

Statistical analyses were performed using IBM SPSS^®^ Statistics software (version 25.0 (IBM Corp., SPSS Inc., NY, USA) (p = 0.05).

## Results

Initially, 71 participants were included, 60 of whom met the eligibility criteria. There was sample loss of 17 at T6 (n = 43) and 22 at T12 (n = 38). The mean age of the participants at T0 was 34.8 years (± 10.29), with 32 females (33.9 ± 10.06 years) and 11 males (37.5 ± 10.98 years) (Figure).

**Figure:**
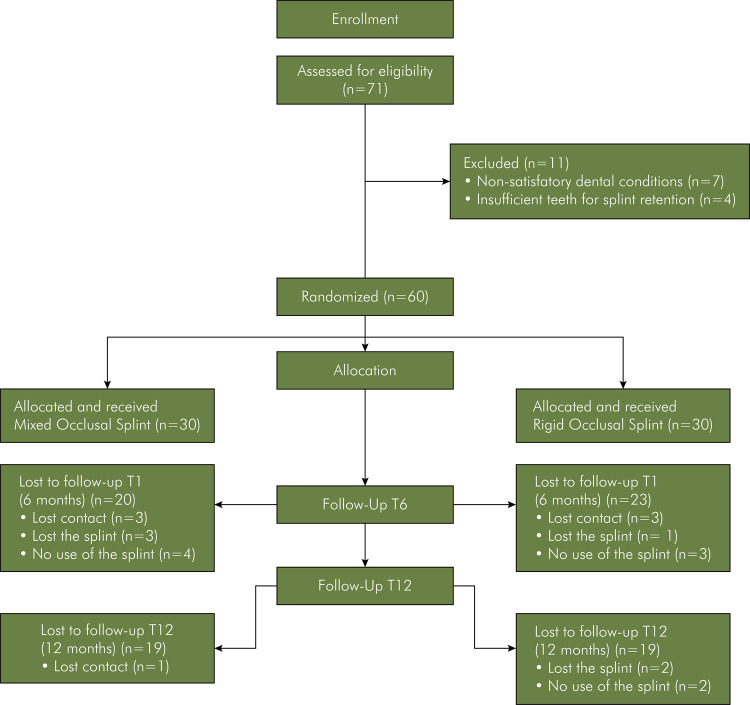
Flowchart summary of sample allocation and sample losses and the times studied (6 and 12 months)

The mean age of the MOS group was 35.8 years (± 10.7) and that of the ROS group was 34.0 years (± 10.1). No dependence was observed between sex and group (p = 0.536) and no difference in age was observed between the two groups (p = 0.688).

When analyzing the MOS and ROS groups individually over time, it was verified that the physical and environmental domains of QoL increased over time, and pain intensity and the frequency of tongue indentation and masseter muscle pain decreased (p < 0.05) (Tables 1 and 2). In the ROS group, the environmental domain of QoL increased over time, and the pain intensity and frequency of masseter muscle pain decreased (p < 0.05) (Tables 1 and 2).

**Table 1:** Descriptive statistics of continuous variables analyzed according to group and time.

Variable	Groups	T0	T6	T12

		Mean ± SD	Mean ± SD	Mean ± SD
QoL Physical*	MOS	3.79 ± 0.61 aA	3.97 ± 0.39 aA	4.02 ± 0.51 aA
	ROS	4.06 ± 0.37 aA	4.14 ± 0.38 aA	4.03 ± 0.45 aA
QoL Psychological*	MOS	3.68 ± 0.47 aA	3.90 ± 0.41 aB	3.89 ± 0.49 aB
	ROS	3.94 ± 0.40 aA	3.88 ± 0.48 aA	3.79 ± 0.35 aA
QoL Social	MOS	3.80 ± 0.73 aA†	3.93 ± 0.61 aA†	3.91 ± 0.91 aA‡
	ROS	3.84 ± 0.73 aA†	3.96 ± 0.65 aA†	3.95 ± 0.61 aA‡
QoL Environment*	MOS	3.53 ± 0.48 aA	3.68 ± 0.58 aAB	3.78 ± 0.46 aB
	ROS	3.68 ± 0.54 aA	3.74 ± 0.49 aA	3.86 ± 0.35 aB
QoL Self-evaluation**	MOS	3.90 ± 0.64 aA	4.05 ± 0.69 aA	4.16 ± 0.50 aA
	ROS	3.91 ± 0.51 aA	4.00 ± 0.43 aA	3.84 ± 0.50 aA
QoL Satisfaction with health**	MOS	3.55 ± 0.94 aA	4.00 ± 0.79 aA	3.79 ± 0.92 aA
	ROS	3.78 ± 0.67 aA	4.04 ± 0.56 aA	3.89 ± 0.66 aA
Mean QoL*	MOS	3.71 ± 0.50 aA	3.92 ± 0.41 aB	3.93 ± 0.53 aB
	ROS	3.87 ± 0.37 aA	3.96 ± 0.38 aA	3.89 ± 0.38 aA
Pain Intensity	MOS	5.50 ± 3.32 aA†	3.05 ± 3.25 aB‡	2.53 ± 2.65 aB‡
	ROS	4.09 ± 3.01 aA†	2.00 ± 2.50 aB‡	0.95 ± 1.61 aB‡
Anxiety**	MOS	0.70 ± 0.66 aA	0.50 ± 0.89 aA	0.37 ± 0.68 aA
	ROS	0.78 ± 0.90 aA	0.87 ± 0.81 aA	0.95 ± 0.91 aA
Depression**	MOS	0.35 ± 0.59 aA	0.20 ± 0.41 aA	0.21 ± 0.42 aA
	ROS	0.13 ± 0.34 aA	0.17 ± 0.39 aA	0.05 ± 0.23 aA
Time of Use**	MOS	-	5.00 ± 2.13 aA	5.21 ± 2.25 aA
	ROS	-	5.35 ± 1.99 aA	4.68 ± 2.24 aA

SD: standard deviation; QoL: quality of life; MOS: mixed occlusal splint; ROS: rigid occlusal splint.

MOS: T0 (n = 20); T6 (n = 20); T12 (n = 19). ROS: T0 (n = 23); T6 (n = 23); T12 (n = 19).

The same lowercase letters indicate no statistically significant differences between groups. *Student’s *t*- parametric test for independent samples; **Non-parametric Mann–Whitney U test for independent samples: p < 0.05.

The same capital letters indicate no statistically significant difference between times, within each group. Time of Use - non-parametric Wilcoxon test for paired samples; other variables - Friedman ANOVA non-parametric test for paired samples and Friedman pairwise multiple comparisons non-parametric test: p < 0.05

**Table 2:** Cross-frequency and p-value according to Group and Time.

Variable	Time	Statistics	YES			p-value

			Group		Total	

			MOS	ROS		
Tongue indentation	0	Score	11aA	7aA	18	0.103
		% group	0.55	0.30	0.42	
	6	Score	6aB	7aA	13	0.975
		% group	0.30	0.30	0.30	
	12	Score	5aB	4aA	9	0.703
		% group	0.26	0.21	0.24	
Buccal indentation	0	Score	13aA	17aA	30	0.526
		% group	0.65	0.74	0.70	
	6	Score	12aA	17aA	29	0.331
		% group	0.60	0.74	0.67	
	12	Score	10aA	11aA	21	0.744
		% group	0.53	0.58	0.55	
Labial indentation	0	Score	0aA	1aA	1	0.259
		% group	0.00	0.04	0.02	
	6	Score	1aA	1aA	2	0.919
		% group	0.05	0.04	0.05	
	12	Score	1aA	0aA	1	0.235
		% group	0.05	0.00	0.03	
TMJ Pain	0	Score	9aA	7aA	16	0.324
		% group	0.45	0.30	0.37	
	6	Score	4aA	3aA	7	0.538
		% group	0.20	0.13	0.16	
	12	Score	4aA	2aA	6	0.374
		% group	0.21	0.11	0.16	
Masseter pain	0	Score	18aA	13bA	31	0.015
		% group	0.90	0.57	0.72	
	6	Score	10aB	7aB	17	0.191
		% group	0.50	0.30	0.40	
	12	Score	6aB	3aB	9	0.252
		% group	0.32	0.16	0.24	
Temporal pain	0	Score	4aA	5aA	9	0.889
		% group	0.20	0.22	0.21	
	6	Score	1aA	3aA	4	0.365
		% group	0.05	0.13	0.09	
	12	Score	2aA	1aA	3	0.547
		% group	0.11	0.05	0.08	
Splint wear	6	Score	6aA	1aA	7	0.023
		% group	0.30	0.04	0.16	
	12	Score	2aA	0aA	2	0.146
		% group	0.11	0.00	0.05	

ROS: rigid occlusal splint; MOS: mixed occlusal splint.

Pearson’s Chi-Square Test: p-value > 0.05 indicates independence between the variable and the group; different lowercase letters indicate a statistically significant difference between the percentages in group for each category of the dependent variable: p < 0.05.

The same capital letters in the column indicate no statistically significant difference between times, for the Yes category, within each group. Splint Wear - non-parametric McNemar’s test; other variables - non-parametric Cochran’s Q test: p < 0.05.

In the analysis of the MOS and ROS groups (Tables 1 and 2), a difference was observed only for splint wear, with a higher frequency of wear at T6 in the MOS group (30%) (p < 0.05). Thus, the description of the results of each evaluation, as well as an analysis of the behavior of the variables over time, was performed using the entire sample, as presented below.

### Primary outcomes

#### Orofacial pain

At T0, most individuals experienced pain in the masseter muscle (72.1%), followed by the TMJ region (37.2%) and temporal muscle (20.9%). A higher frequency of TMJ pain was observed at T0 (37.2%) than at T6 (16.2%) (p < 0.05) (Table 3).

**Table 3:** Cross-frequency and p-value for lingual, buccal, and labial indentation; TMJ, masseter and temporal pain and splint wear.

Variable	T0	T6		p-value	T0	T12		p-value	T6	T12		p-value
		
		no	yes			no	yes			no	yes	
Tongue indentation	no	23	2	0.180	no	21	2	0.109	no	25	4	1.000
	yes	7	11		yes	8	7		yes	4	5	
Buccal indentation	no	11	2	1.000	no	10	2	0.180	no	12	1	0.219
	yes	3	27		yes	7	19		yes	5	20	
Labial indentantion	no	40	2	1.000	no	36	1	1.000	no	35	1	1.000
	yes	1	0		yes	1	0		yes	2	0	
TMJ pain	no	27	0	0.004*	no	21	3	0.057	no	29	3	1.000
	yes	9	7		yes	11	3		yes	3	3	
Masseter pain	no	11	1	0.001*	no	8	1	0.000*	no	21	2	0.109
	yes	15	16		yes	21	8		yes	8	7	
Temporal pain	no	32	2	0.180	no	30	1	0.219	no	34	2	1.000
	yes	7	2		yes	5	2		yes	1	1	
Splint wear	no	-	-	-	no	-	-	-	no	32	1	0.375
	yes	-	-		yes	-	-		yes	4	1	

Non-parametric test of the significance of McNemar changes

* p-value < 0.05 indicates difference between times

Moderate pain was observed at T0 and T6, and mild pain was observed at T12; the pain was higher at T0 (4.74 ± 3.20) than at T6 (2.49 ± 2.89) and T12 (1.74 ± 2.31) (p < 0.05) (Table 4).

**Table 4:** Descriptive statistics for quality of life, pain intensity, anxiety, depression, and time of use.

Variable	T0 x T6	T0 x T12	T6 x T12

	Mean ± SD	Mean ± SD	Mean ± SD
QoL physical*	3.94 ± 0.52 a	3.91 ± 0.51 a	4.03 ± 0.39 a
	4.06 ± 0.39 b	4.03 ± 0.47 a	4.03 ± 0.47 a
QoL psychological*	3.28 ± 0.45 a	3.77 ± 0.45 a	3.86 ± 0.45 a
	3.89 ± 0.44 a	3.84 ± 0.42 a	3.84 ± 0.42 a
QoL social*	3.82 ± 0.72 a	3.73 ± 0.70 a	3.90 ± 0.63 a
	3.95 ± 0.62 a	3.93 ± 0.76 a	3.93 ± 0.76 a
QoL environment*	3.61 ± 0.51 a	3.53 ± 0.47 a	3.64 ± 0.52 a
	3.72 ± 0.53 a	3.82 ± 0.41 b	3.82 ± 0.41 b
Self-perceived QoL**	3.91 ± 0.57 a	3.91 ± 0.57 a	4.02 ± 0.56 a
	4.02 ± 0.56 a	4.00 ± 0.52 a	4.00 ± 0.52 a
QoL satisfaction environment with health**	3.67 ± 0.81 a	3.67 ± 0.81 a	4.02 ± 0.67 a
	4.02 ± 0.67 b	3.84 ± 0.79 a	3.84 ± 0.79 a
Mean QoL*	3.80 ± 0.44 a	3.73 ± 0.42 a	3.90 ± 0.38 a
	3.94 ± 0.39 b	3.91 ± 0.45 b	3.91 ± 0.45 a
Pain intensity**	4.74 ± 3.20 a	4.79 ± 3.17 a	2.34 ± 2.81 a
	2.49 ± 2.89 b	1.74 ± 2.31 b	1.74 ± 2.31 a
Anxiety**	0.74 ± 0.79 a	0.74 ± 0.79 a	0.70 ± 0.86 a
	0.70 ± 0.86 a	0.66 ± 0.85 a	0.66 ± 0.85 a
Depression**	0.23 ± 0.48 a	0.23 ± 0.48 a	0.19 ± 0.39 a
	0.19 ± 0.39 a	0.13 ± 0.34 b	0.13 ± 0.34 a
Time of use*	-	-	5.05 ± 2.09 a
	-	-	4.95 ± 2.23 a

QoL: Quality of Life.

*Student’s *t*-test for paired samples: p < 0.05; **Wilcoxon’s non-parametric test for paired samples: p < 0.05.

Different letters indicate statistically significant differences between times

T0xT6 (n = 43); T0xT12 (n = 38); T6xT12 (n = 38)

## Quality of life

The mean QoL was classified as regular at the three time points. When comparing the QoL variables between times, it was noticed that physical QoL at T6 (4.06 ± 0.39) was higher than at T0 (3.94 ± 0.52). Environmental QoL was higher at T12 (3.82 ± 0.47) than at T0 (3.53 ± 0.47) and T6 (3.64 ± 0.52). Mean QoL was higher at T6 (3.94 ± 0.39) than at T0 (3.80 ± 0.44), and higher at T12 (3.91 ± 0.45) than at T0 (3.73 ± 0.42). QoL satisfaction with health was higher at T6 (4.02 ± 0.67) than at T0 (3.67 ± 0.81) (p < 0.05) (Tables 1, 4).

## Secondary outcomes

### Indentations

Indentations in the buccal mucosa were most frequent at T0 (69.76%), followed by lingual (34.88%) and labial (2.32%) indentations (p < 0.05) (Table 3).

### Anxiety and depression

Anxiety and depression were absent during the three periods. Over time, there was a lower depression score at T12 (0.13 ± 0.34) than at T0 (0.23 ± 0.48) (p < 0.05) (Tables 1,4).

### Splint wear

At T6, there was more splint wear in the MOS group (five splints) than in the ROS group (one splint) (p < 0.05). At T12, wear was observed in one MOS and one ROS (p > 0.05) (Tables 2, 3).

The rate of wear of the splints was 16.3% at T6 and 5.3% at T12, with no significant difference between the time points (p > 0.05). The average duration of device use was 5 days/week (Tables 1 and 4).

The other variables and analyzed times did not show any differences over time (p > 0.05).

## Discussion

In the sample studied, the MOS and ROS groups were similar in the clinical and psychological aspects studied, except for splint wear, which had a higher occurrence in the MOS group. Therefore, comparisons over time were also made with the data from both groups combined, and improvements in pain and QoL scores were observed, showing that both types of splints were adequate for controlling SB signs and symptoms.

In the current study, higher levels of pain were observed at the beginning of the trial and decreased throughout the study in the three regions evaluated; this decrease was statistically significant in the masseter muscle and TMJ region from T0 to T6. This result supports the statement that occlusal splints can reduce facial pain^21^ because their use reduces the activity of the masseter^23^ and the anterior part of the temporal muscle,^24^and improves TMJ pain.^25^ Pain intensity decreased over time, which is supported by a study that found a reduction in pain intensity and the number of sore muscles in patients with myofascial pain after 6 weeks of occlusal splint therapy.^26^ Pain improvement may also be attributed to the placebo effect while using these splints.^27^

In this study, the splints were worn for over 12 months, five days per week. The frequency of splint use is a key factor, as they can reduce SB episodes after three months,^9^ and their non-use for 15 consecutive days can aggravate painful symptoms.^28^ It has been reported that after eight weeks of use, there is no substantial inhibition of the motor activity of the masticatory muscles.^29^ Although there is a lack of evidence on the effects of splints on muscle activity, there is a consensus that they protect the teeth from excessive wear in patients with SB.^29^

In the current study, both splints promoted a decrease in painful symptoms and pain intensity in the masseter muscle and TMJ region and improved QoL scores. It is noteworthy that the greatest change occurred in the physical domain of QoL, which improved from “regular” at T0 to “good” at T6. This demonstrates the importance of controlling SB, especially in terms of physical aspects such as pain, discomfort, sleep, and rest. These results are supported by a study in which occlusal splints led to an improvement in QoL domains in individuals with SB.^30^

Indentations in the buccal mucosa are clinical signs of tooth clenching,^5^ and lingual indentations are clear indicators of bruxism.^5^ In this study, indentation frequency was highest in the buccal mucosa, followed by the tongue. Tongue indentation decreased over time in the MOS group; however, when the groups were analyzed together, the frequencies of the three types of indentations did not decrease statistically. During SB activity, muscle strength is greater than during normal function.^10^ Thus, the force exerted by the tongue against the teeth during SB possibly also increases, which may be responsible for trauma and ulcerations of the tongue and buccal mucosa.^31^ As the use of an occlusal splint reduces the pressure on the tongue,^32^a reduction in that frequency was expected; however, this did not occur.

Associations between anxiety and the state of arousal and increased activation of the sympathetic system can affect motor responses;^33^ considering the correlations between these factors, a questionnaire was administered to verify the presence of anxiety. However, most participants in this study did not exhibit this behavior. Similarly, none of the evaluated individuals had depression. However, the occurrence of these conditions in individuals with bruxism remains controversial. One study found no association between the intensity of SB and the degree of depression.^34^ On the contrary, another study found increased frequency and severity of depressive symptoms in patients with bruxism.^35^Thus, considering that psychological factors and SB may have normal fluctuations,^36^further studies are needed to prove this cause-and-effect relationship.^34^Specifically, assessments of the same individuals at different times are necessary.

Among the types of occlusal splints, we chose to use stabilizers that cover all teeth,^10^ installed in the upper dental arch. The ROS was selected as the gold standard as it is the most suitable for controlling bruxism.^13^It is considered effective^12^ as it has an occlusal surface hard enough not to be damaged,^37^ allows for occlusal balance to be achieved in the long term,^15^ and has good adaptive potential.^37^

When preparing the mixed splints, we inserted a single resin rod into the acetate sheet to reduce working time. To make the rigidity closer to that of the rigid splint, the acetate sheet was worn at the cusp tips to promote contact between the acrylic resin and the teeth. We observed that this splint had a higher rate of wear than the rigid splint, as found in an *in vitro* study.^38^ This may have occurred because the chemically activated acrylic resin absorbs a large amount of oral fluids^39^ and undergoes greater wear over time because of its lower degree of polymerization and higher concentration of residual monomers. This causes the maximum strength and stiffness to be lower than that of the thermally-activated acrylic resin^40^ used in ROS.

On the other hand, the lower rigidity of the chemically activated resin can act as an advantage, as it causes less wear in enamel, dentin, and various types of composite resin.^38^ Meanwhile, rigid splints are more suitable for use in individuals who have little or no dental wear or whose teeth can be restored with more rigid materials.^38^ In addition, the production of MOS does not require molding of the antagonist arch, assembly in an articulator, or sending to a dental technician, thus reducing the cost of its production.^10^

Although the MOS exhibited more wear and was less resistant, it was considered adequate because it did not need to be replaced when wear was observed. In addition, it can be used immediately after oral rehabilitation, minimizing the possibility of damage to restorations and prostheses,^10^ because a dental surgeon can make the splint during the same appointment.

Based on the results of this study, the mixed splint proved adequate for controlling the effects of SB. Despite the low wear resistance of this device, it is beneficial because of its low cost and reduced wear in dental tissues and composite resin restorations. These advantages allow us to suggest the MOS as a viable option for widespread use, especially in public health services providing benefits to low-income populations.

The main limitations of the present study were that a definitive diagnosis of SB was not obtained, the evaluation of splint wear was qualitative, it was impossible to blind the operators, and the number of hours of splint use was not confirmed. Further quantitative studies should be conducted using appropriate methodologies to overcome these limitations.

## Conclusions

The mixed occlusal splint showed a higher proportion of wear than the rigid splint but had similar results to the rigid splint in reducing the frequency and intensity of pain in the masseter muscle and TMJ and in improving the quality of life scores. Thus, the use of a mixed splint seems to be effective in controlling the signs and symptoms of SB.
